# Novel Rat Model of Repetitive Portal Venous Embolization Mimicking Human Non-Cirrhotic Idiopathic Portal Hypertension

**DOI:** 10.1371/journal.pone.0162144

**Published:** 2016-09-02

**Authors:** Sabine Klein, Christian Hinüber, Kanishka Hittatiya, Robert Schierwagen, Frank Erhard Uschner, Christian P. Strassburg, Hans-Peter Fischer, Ulrich Spengler, Jonel Trebicka

**Affiliations:** 1 Department of Internal Medicine I, University of Bonn, Bonn, Germany; 2 Institute of Pathology, University of Bonn, Bonn, Germany; 3 Faculty of Health Sciences, University of Southern Denmark, Odense, Denmark; University of Navarra School of Medicine and Center for Applied Medical Research (CIMA), SPAIN

## Abstract

**Background:**

Non-cirrhotic idiopathic portal hypertension (NCIPH) is characterized by splenomegaly, anemia and portal hypertension, while liver function is preserved. However, no animal models have been established yet. This study assessed a rat model of NCIPH and characterized the hemodynamics, and compared it to human NCIPH.

**Methods:**

Portal pressure (PP) was measured invasively and coloured microspheres were injected in the ileocecal vein in rats. This procedure was performed weekly for 3 weeks (weekly embolization). Rats without and with single embolization served as controls. After four weeks (one week after last embolization), hemodynamics were investigated, hepatic fibrosis and accumulation of myofibroblasts were analysed. General characteristics, laboratory analyses and liver histology were collected in patients with NCIPH.

**Results:**

Weekly embolization induced a hyperdynamic circulation, with increased PP. The mesenteric flow and hepatic hydroxyproline content was significantly higher in weekly embolized compared to single embolized rats (mesenteric flow +54.1%, hydroxyproline +41.7%). Mesenteric blood flow and shunt volumes increased, whereas splanchnic vascular resistance was decreased in the weekly embolization group. Fibrotic markers αSMA and Desmin were upregulated in weekly embolized rats.

**Discussion:**

This study establishes a model using repetitive embolization via portal veins, comparable with human NCIPH and may serve to test new therapies.

## Introduction

Non-cirrhotic idiopathic portal hypertension (NCIPH) is a poorly understood disease of varied etiology, which leads to portal hypertension and its complications. In the absence of cirrhosis, other synonyms are non-cirrhotic portal fibrosis (NCPF), or hepatoportal sclerosis and obliterative venopathy in Eastern countries [1]. Importantly, this condition occurs in the absence of other well characterized causes of portal hypertension such as chronic liver disease and splanchnic venous thrombosis [2–5]. The prognosis of this disease is considered as benign with a 5 year survival of almost 100%, but long term outcomes are still unknown [4,6,7]. Since the prevalence of NCIPH in the Western world is low, recently published literature is rare [8]. Various conditions such as chronic infectious diseases, toxin or drug exposure, genetic and immunological disorders have been determined as potential causes of NCIPH [3,6].

Usually the first symptoms of NCIPH are splenomegaly and pancytopenia [1,8]. Gastro-oesophageal varices and bleeding complications remain a major burden for NCIPH-patients. Therefore, prophylaxis and treatment of variceal bleeding are of clinical relevance [4,7].

However, to test new treatment options, experimental models are still missing. One reason for the absence of experimental models is that the underlying pathomechanisms of NCIPH have been poorly understood so far [3,9,10]. In liver samples of patients with NCIPH, vascular lesions in the portal venules have been observed [3]. Micro-thrombotic lesions in small portal venules have been proposed as the initial cause of NCIPH [10].

Therefore, we studied the role of micro-thrombotic lesions as a possible initial step for the processes leading to NCIPH in a new rat model using microsphere embolization and compared the findings with human NCIPH.

## Material and Methods

### Patients

Five patients with portal hypertension and absence of other causes of hepatic fibrosis or cirrhosis were investigated in the study in the Department of Internal Medicine I, University of Bonn, Germany. Their histology was compared to five non-cirrhotic liver samples without portal hypertension. The use of human liver samples was approved by the Human Ethics Committee of the University of Bonn (Ethikkommission der Universität Bonn, reference number 029/13). It was not possible to obtain written or oral consent, because patients data and pathological specimen were analyzed retrospectively using remnant tissue specimens. Also patients had been lost for follow up and could no longer be contacted. In line with our ethical regulations it is allowed to use retrospective data in anonymous form, when the patients can no longer be contacted. The recruitment period was from 2009 to 2014 and routine laboratory values from baseline and follow up were observed. Biochemical blood analyses were performed using standard tests. A liver biopsy was taken from all patients and evaluated by two senior pathologists. Parenchyma, vascularization and biliary vessels were evaluated.

#### Ultrasound examination

Ultrasound examination was performed in all patients by senior internists applying current standards. Ultrasound included evaluation of liver parenchyma (fibrotic, nodular regenerative hyperplasia (NRH) and atrophic; hyperchoic and hypoechoic; homogeneous and heterogeneous), evaluation of liver vascularization (normal and abnormal; downsized and enlarged; regular limited and irregular limited), the evaluation of biliary ducts (enlarged and downsized) and the portal vein (obstructed, unobstructed).

#### Sirius red staining and αSMA immunohistochemistry of human biopsies

For the detection of collagen fibers, liver specimen were fixed in 10% formalin, paraffin-embedded and stained in 0.1% Sirius-red in saturated picric acid (Chroma, Münster, Germany) using standard methods as previously described [11].

For immunohistochemical (IHC) staining of α-smooth muscle actin (αSMA), slides with sections were incubated with a mouse-anti-αSMA (clone 1A4; Sigma–Aldrich, St. Louis, USA) diluted 1:100 in Tris–buffered saline overnight. A secondary biotinylated rabbit-anti-mouse antibody, absorbed with rat serum (Dako, Glostrup, Denmark), was subsequently applied (1:300, 30 min) and complexed with streptavidin-conjugated alkaline phosphatase (1:500, 30 min; Dako). Finally, slides were developed with AEC (3-amino-9-ethylcarbazole) (15 min; Dako) and counterstained with hematoxylin. The amount of staining was evaluated by computational analysis (Histoquant; 3DHistech, Budapest, Hungary). Quantification (% of stained area) of IHC staining is expressed as mean±SEM.

#### Human CD105 staining

For immunohistochemical staining of hepatic CD105, liver specimens were fixed in 10% formalin, paraffin-embedded and stained with goat anti-human endoglin/CD105 (R&D Systems, Inc. Canada) at 4°C overnight. Tissue was stained using anti-goat HRP-DAB and counterstained with hematoxylin. Staining was evaluated by Pannoramic Viewer (Histoquant; 3DHistech, Budapest, Hungary) and quantification (% of stained area) of IHC staining is expressed as mean±SEM.

### Animals

We used 23 Sprague-Dawley rats for our experiments. Six week-old male Sprague-Dawley rats were housed in a 12:12 h light-dark cycle, with controlled temperature (21°C ± 2°C), and *ad libitum* standard rat chow and water. The responsible committee for animal studies in North Rhine-Westphalia approved the study (Landesamt für Natur und Umwelt, LANUV 84–02.04.2014.A137).

#### Assessment of hepatic fibrosis

In corresponding segments (200mg) of snap-frozen rat livers, the hepatic hydroxyproline content was determined photometrically as described previously [11,12].

#### Hepatic αSMA staining in rat

Immunohistochemical stainings for hepatic αSMA were prepared using a cryostat. Cryo sections (4–6μm) of snap-frozen liver samples were fixed and incubated with mouse-anti-αSMA antibody (Sigma Aldrich, München, Germany). Thereafter, a biotinylated donkey-anti-rat secondary antibody was used (Abcam, Cambridge, UK). Sections were detected and quantified using computerized image capture device (Histoquant; 3DHistech, Budapest, Hungary). Results are expressed as mean±SEM.

#### Protein expression measurement

Liver pieces of all rats (weekly embolization, single embolization and control) were collected after performing the microsphere technique and snap-frozen. Western blot analyzes were performed with rabbit anti-αSMA antibody (Sigma Aldrich, Germany) and for Desmin with rabbit anti-Desmin antibody (GeneTex Inc., Irvine, CA, USA). Thereafter, the membranes were incubated with corresponding secondary peroxidase-coupled antibodies (Calbiochem, San Diego, USA). The housekeeping gene GADPH was used as loading control. Blots were developed with enhanced chemiluminescence. Intensities of the digitally detected bands were evaluated densitometrically using Chemi-Smart, normalized to control rat livers and represented as mean±SEM.

#### Portal pressure measurements and induction of portal hypertension in rats

Rats were anaesthetized by intraperitoneal (i. p.) injection of ketamine/xylazine (78 mg/kg / 12.5 mg/kg). First a laparotomy was performed, a catheter was inserted in an ileocecal vein and pushed to the portal vein to measure the PP. After PP measurement, weekly embolized rats (n = 5) received every week 150.000 coloured microspheres (violet (week 1), blue (week 2), yellow (week 3); 15μm diameter, Triton-Technologies, San Diego, USA) via the catheter in the ileocecal vein. The single embolized rat group (n = 8) received 150.000 microspheres (blue) once after the first PP measurement. The control group (n = 10) did not receive any microspheres after PP measurements. After weekly PP measurements ileocecal veins were ligated and the linea alba and skin were closed with 4–0 Vicryl and 3–0 Prolene, respectively. All surgeries were performed under aseptic conditions. To prevent pain after assessment of the PP and microsphere injection rats received Carprofen (5mg/kg/d;) s. c. for at least 5 days as described previously [13]. The early/human endpoints were defined the following characteristics of the rats: Immobility, isolation from other rats, walking on tiptoe or crookedly, scrubby fur, even breathing, to malnourished, insufficient liquid intake, body weight and the percent to the initial body weight, body temperature, inflamed eyes or interface, swollen abdomen, tremulousness and diarrhea. Stockmen score each characteristic for each rat every day. At a defined scoring point level rats will be euthanized immediately by carbon dioxide intoxication. The direct euthanasia is performed when rats lose 20% of their initial body weight, if the rats is unable to move, if the rat has no liquid intake anymore, if the rat is cachectic or if the feces is fluid. In the experimental group of “weekly embolized rats” 50% of the rats were euthanized prior the experimental endpoint due to weight lost and inflamed interfaces. The same reasons caused the euthanasia of 20% in the “single embolization” group before the experimental endpoint. In the control group all rats reached the experimental endpoint, as expected.

#### Portal and systemic hemodynamic assessment

Hemodynamic studies were performed under ketamine/xylazine anesthesia (78mg/kg / 12.5mg/kg i.p.) as previously described [14–17]. Rats were fasted overnight but allowed free access to water. Median laparotomy was performed; a PE-50 catheter was introduced into a small ileocecal vein and advanced to the portal vein for the measurement of PP. The left femoral artery was cannulated with a PE-50 catheter for measurement of the mean arterial pressure (MAP) and blood withdrawal. Via the right carotid artery, another PE-50 catheter was advanced into the left ventricle under pulse curve control. This catheter was used for microsphere application to investigate the cardiac output. The catheters in the femoral artery and the portal vein were connected to a pressure transducer (ADInstruments Ltd, Oxford, United Kingdom) for blood pressure measurement. The zero point was 1 cm above the operating table. After insertion of all catheters, rats were allowed to stabilize hemodynamically for 30 min [14].

#### Microsphere technique

Hemodynamics were investigated using the coloured microsphere technique as previously described [14–17]. The colored microsphere technique was validated by the more frequently used radioactive microsphere method [18]. It has the advantage of being non-radioactive and using different colors at the same organism. This technique has been shown very accurate in the measurement of hemodynamics in many studies of our group and others [14–16,18–23]. A reference sample was obtained for 1 min at a rate of 0.65mL/min, using a continuous withdrawal pump (Hugo-Sachs-Elektronik,March-Hugstetten,Germany). 300,000 systemic (yellow) microspheres were suspended in 0.3mL saline containing 0.05% Tween and injected in the left ventricle 10 sec after the withdrawal pump had been started. Mesenteric portal-systemic shunt volume was estimated after injection of 150,000 white microspheres in 0.3 ml saline containing 0.05% Tween in an ileocecal vein within 30 seconds [24]. At the end of hemodynamic measurements the catheter in the ventricle is used to inject an overdose of ketamine (1ml of 10%) which euthanizes rats immediately without being afflicted with pain. The blood reference probe was digested by addition of 3.8 ml 5.3 M KOH and 0.5 ml Tween 80 and subsequent boiling for 1 hour. The digested tissues and blood samples were vortexed and filtered using Whatman Nucleopore filters (Whatman International Ltd.,Madison,UK). The colour of the filtered microspheres was dissolved in 0.2 ml dimethyl-formamide, and the absorption was measured using spectrophotometry. Splanchnic vascular resistance was calculated from the ratio between splanchnic perfusion pressure and splanchnic blood flow, without including hepatic arterial flow. Mesenteric shunt flow was measured as the fraction of white microspheres in the lung from total white microspheres injected in an ileocolic vein.

#### Statistical analysis

Clinical data were collected and evaluated with SPSS statistical analysis software (IBM SPSS Statistics for Windows, Version 22.0, released 2013. Armonk, NY: IBM Corp.). Data are presented as median, maximum and minimum and mean±SEM. Data of laboratory values at the point of first contact were compared with data at the point of follow up in all groups. In all experimental animals the Wilcoxon test was used for comparison within one group. Unpaired data were compared by the Mann-Whitney test. Data are presented as mean±SEM. P values <0.05 were considered statistically significant.

## Results

### Clinical characteristics of NCIPH patients

All five examined NCIPH patients were between 21 and 72 years old at baseline with a MELD-score between 6 and 16. All patients had oesophageal varices as a sign of portal hypertension.

Doppler-Sonography showed a patent portal vein in all patients. The liver parenchyma was described as hyperechoic and heterogeneous in four patients. Hepatic veins were irregular limited in five patients and of smaller size in three patients.

### Human liver histology

None of the five liver biopsy specimens of humans had features of cirrhosis. The most common findings were fibrosis in periportal and perisinusoidal areas in two patients (40%). Three patients (60%) showed slight circulatory disturbances of liver perfusion. One patient (20%) had signs of atrophia and four patients (80%) had hyperplasia. Two of the NCIPH patients (40%) showed NPH and signs of abnormal vascularization (Table 1).

**Table 1:** General characteristics of the patients with NCIPH. General characteristics of the patients have been investigated in all patients and are shown in all patients. Characteristics of the parenchyma and of the liver have been evaluated by senior pathologists in specimens of the liver biopsies.

Parameters	IPH patients
**Number of patients** (n)	5
**Gender** (female/male)	(2/3)
**Age at inclusion** (years) median, (range)	54 (21–72)
**MELD-score** median, (range)	9 (6–16)
**Oesophageal varices** (no/yes)	0 / 5
**Biopsy** (no/yes)	0 / 5
**Fibrosis** (no/yes)	3 / 2
**Abnormal vascularisation** (no/yes)	2 / 3
**Atrophia** (no/yes)	4 / 1
**NRH (nodular regenerative hyperplasia)** (no/yes)	3 / 2

### Human hepatic Sirius red stainings

**Fig 1:**
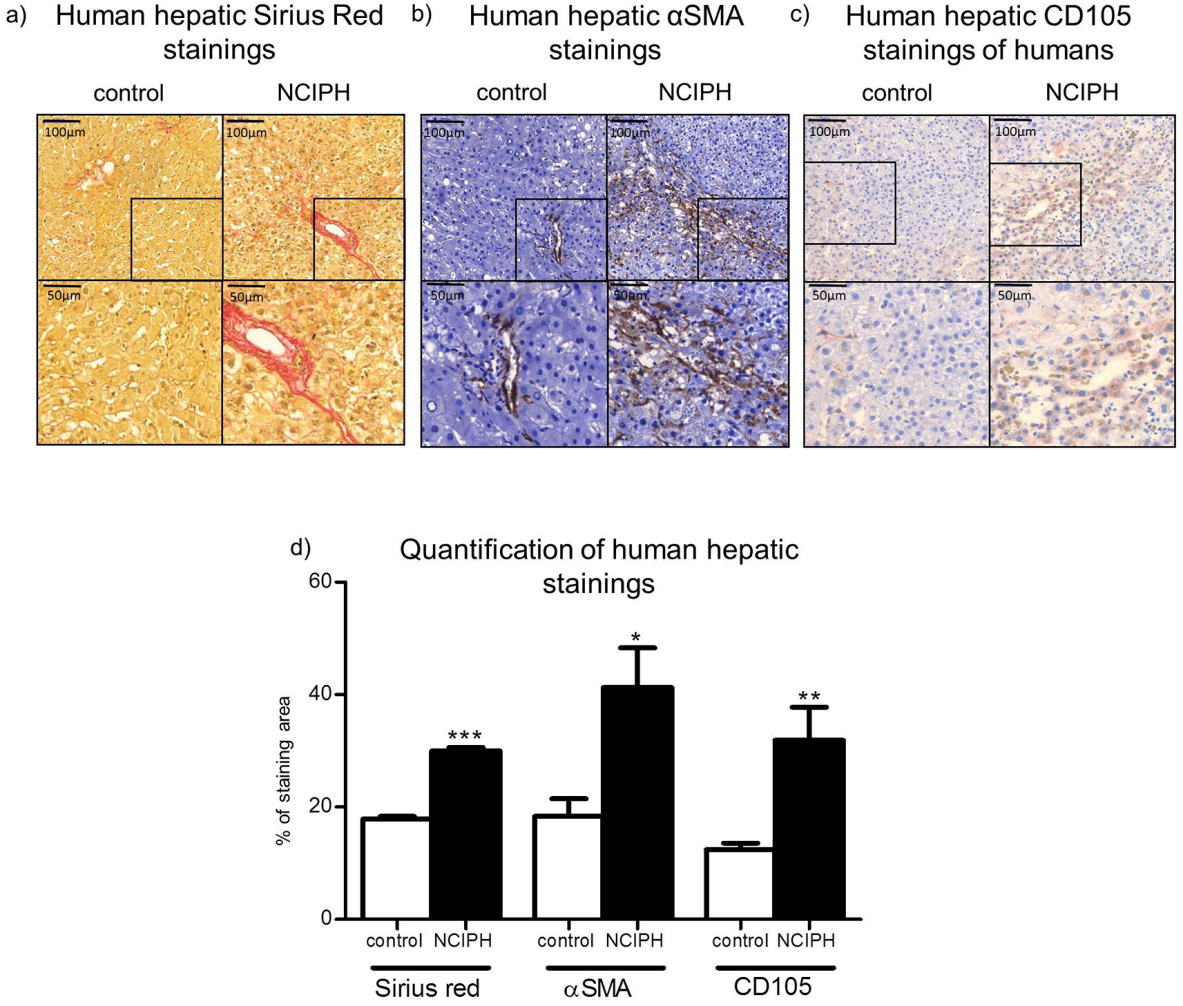
Human liver histology. **a) Human hepatic Sirius Red staining.** Liver specimen of healthy control and NCIPH were stained with Sirius red to detect collagen fibers. The staining of Sirius red was increased in NCIPH patients compared to healthy controls. **b) Human hepatic αSMA staining.** Liver specimen of healthy control and NCIPH were stained with αSMA to detect activated hepatic stellate cells. The staining of αSMA was increased in NCIPH patients compared to healthy controls. **c) Human hepatic CD105 staining.** Liver specimen of healthy control and NCIPH were stained with CD105 to detect endoglin, which is involved in the cytoskeletal organization affecting cell morphology and migration of endothelial cells. The staining of CD105 was increased in NCIPH patients compared to healthy controls. **d) Quantification of human hepatic stainings.** Sirius red, αSMA and CD105 stainings were quantified in human NCIPH liver specimen and compared to healthy controls using computerized image capture (Histoquant; 3DHistech, Budapest, Hungary). All stainings were significantly increased in NCIPH liver specimens compared to healthy controls. */**/***p<0.05/0.001/0.0001.

In the specimens of human NCIPH and controls Sirius red staining was performed to access collagen accumulation. Increased Sirius red staining was observed in NCIPH patients around the portal tract compared to healthy control liver specimens (Fig 1a).

### Human hepatic αSMA stainings

NCIPH patients showed increased hepatic αSMA staining within portal tracts compared to control liver specimens (Fig 1b).

### Human hepatic CD105 staining

The expression of the endothelial marker CD105, also called endoglin, which is involved in angiogenesis and hypoxia, was upregulated in livers of NCIPH patients, shown by immunohistochemically stainings (Fig 1c).

### Quantification of human hepatic stainings

Quantifications of all human hepatic stainings were performed using computerized image capture (Histoquant; 3DHistech, Budapest, Hungary). Hepatic stainings of NCIPH patients were compared with control stainings and expressed as % of stained area. Stainings of αSMA, Sirius red and CD105 were significantly increased in NCIPH liver specimens compared to controls (Fig 1d).

### Rationale of the animal models to mimic human NCIPH

**Fig 2:**
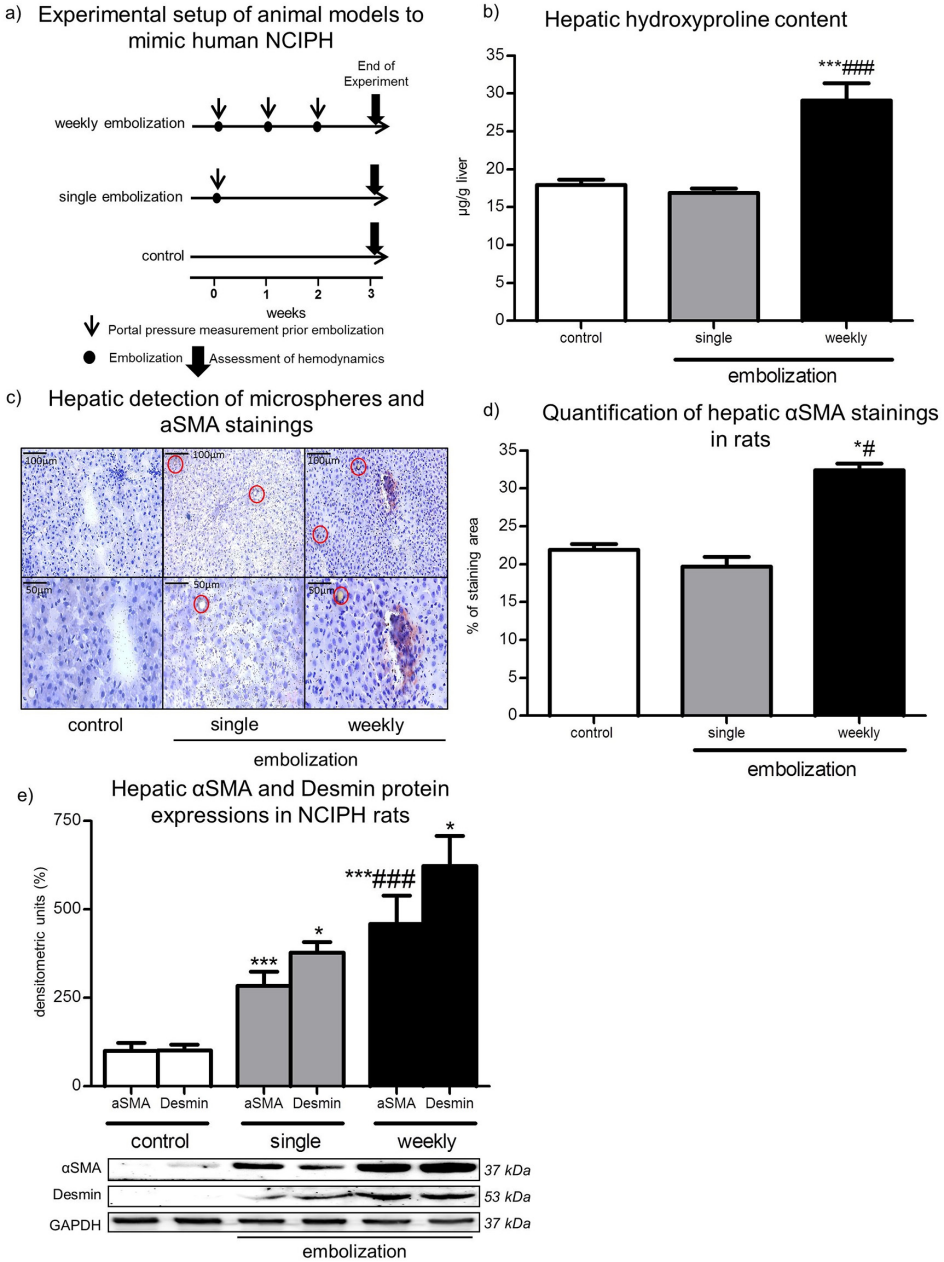
Animal models. **a) Experimental setup of animal models to mimic NCIPH.** The experimental setup of weekly embolized, single embolized and control rats is shown as timeline from start of experiments until the end of experiments after 3 additional weeks. PP measurements are marked as thin arrows and were always performed before embolization, marked by dots. Hemodynamic assessments at the end of experiments are marked as bold arrows. **b) Hepatic hydroxyproline content.** The hydroxyproline content was evaluated in rat livers of all groups at the end of experiment. Livers of weekly embolized rats showed increased hydroxyproline content compared to singe embolized and control rats. The results are shown as hydroxyproline content in μg/g liver. **c) Hepatic αSMA stainings.** After αSMA stainings of all rat livers, most microspheres were found in weekly embolized rat livers, marked with red circles. Fewer microspheres were found in single embolized rat livers, whereas no microspheres were present in livers of control rats. Liver specimens were stained with αSMA to detect activated HSC in rats. The activation of HSC was increased in livers of single embolized but most in rat livers after weekly embolization. **d) Quantification of hepatic αSMA staining in rats.** αSMA stainings were quantified in liver specimens of control, single and weekly embolized rats. The αSMA staining was significantly increased in weekly embolized hepatic specimens compared to single embolized and control liver specimens of rats. **e) Hepatic αSMA and Desmin protein expressions in rats.** The protein expression levels of αSMA and Desmin in livers of control, single and weekly embolized rats were investigated by quantifications of western blots. Weekly embolized rats showed the most expression levels of αSMA and Desmin. All results were normalized to control values. Below, illustration of representative western blots of αSMA and Desmin expression levels in livers of control, single and weekly embolized rats are shown. The housekeeping gene GAPDH was used as loading control. *p<0.05 / **p<0.005 / ***p<0.0001 vs. control; #/###p<0.05/0.0001 vs. single embolization.

We investigated three groups of rats: In weekly embolized rats PP was measured every week for three weeks prior microsphere embolization. PP of single embolized rats was measured invasively every week, but rats were only embolized with microspheres after the first PP measurement. In control rats PP was measured every week for three times but no microsphere injection was performed. At the end of experiments the PP and the MAP were measured invasively and the coloured microsphere technique was performed in all groups of rats to investigate hemodynamics (Fig 2a).

### Hepatic hydroxyproline content

Collagen amount in livers was investigated by the hydroxyproline content. In weekly embolized rats, significantly higher hepatic hydroxyproline content was measured compared to rats after single embolization or to control rats. The single embolization of microspheres in the ileocecal vein did not increase the hepatic hydroxyproline content compared to control rats (Fig 2b).

### Hepatic detection of microspheres and hepatic αSMA stainings

At the end of experiments livers were stained with αSMA to detect activated hepatic stellate cells (HSC) which promote portal hypertension. The microspheres could be detected in hepatic specimens of single and weekly embolized rats (Fig 2c, red circles). Especially signs of atrophia and hyperplasia were observed in the vicinity of microspheres. Increased αSMA stainings and subsequently a higher grade of HSC and myofibroblast activity was detected in weekly embolized rats than in single embolized or in control rats (Fig 2c and 2d).

### Hepatic αSMA and Desmin protein expressions in NCIPH rats

The increased HSC and myofibroblast activity in weekly embolized rats was confirmed by western blot analysis of hepatic αSMA and hepatic Desmin expression levels, as an additional marker for HSC. Even after a single embolization rats expressed significantly more hepatic αSMA and Desmin than control rats. In weekly embolized rats the hepatic protein expression levels of αSMA and Desmin were significantly higher than in control or single embolized rats (Fig 2e).

### Development of portal hypertension in NCIPH rats

**Fig 3:**
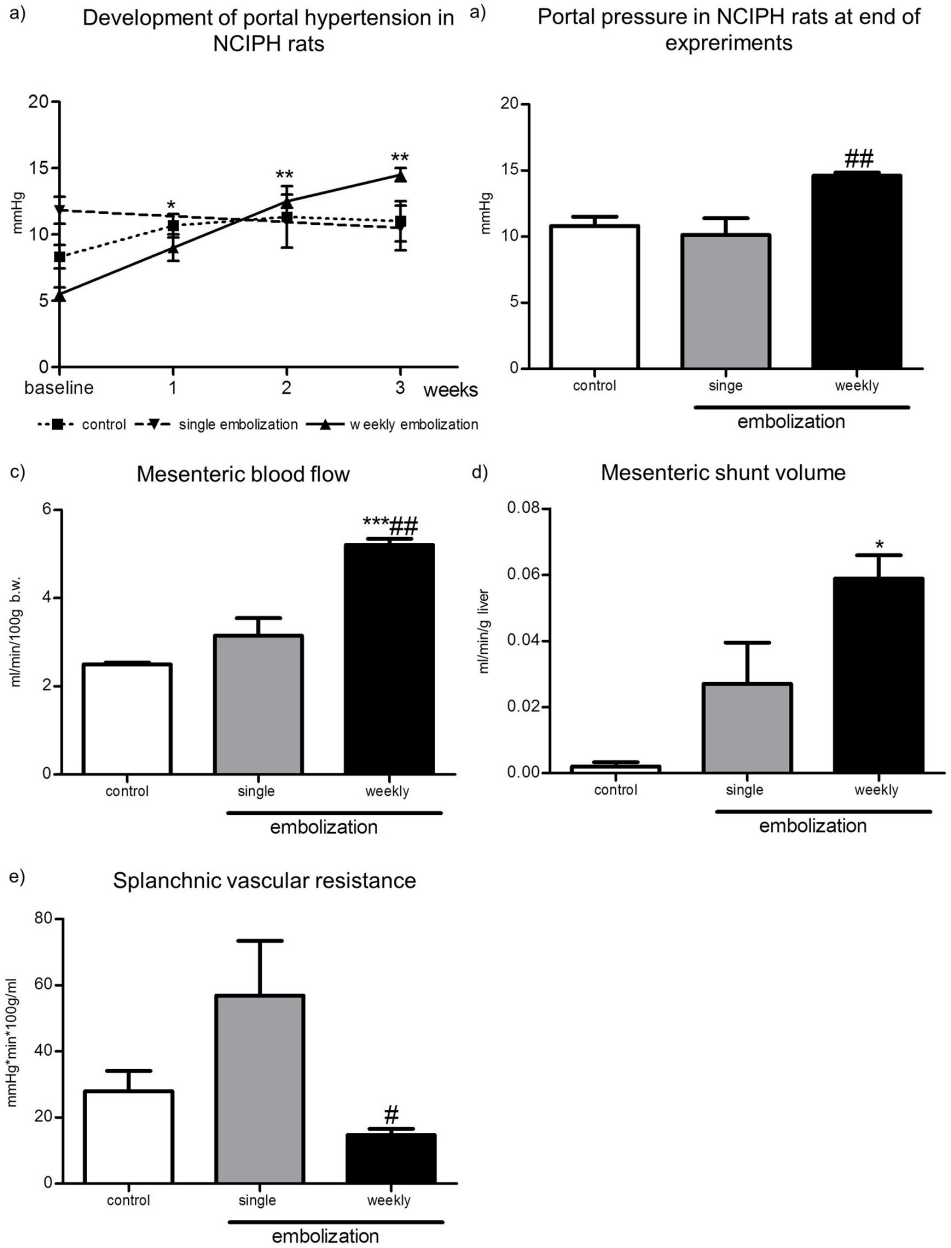
Portal and systemic hemodynamic assessment and the NCIPH model. **a) Portal pressure.** Portal pressure was taken every week in weekly embolized and in control rats. In single embolized rats, the PP was taken at the beginning and the end of experiment. Portal pressures are shown in mmHg. Significant differences are evaluated using paired t-Test within the same group. **b) Portal pressure at the end of experiments.** At the end of experiments, the portal pressures were measured invasively in all rats. After weekly embolization, the PP was significantly higher compared to rats after a single embolization. The portal pressures are shown in mmHg. The significant difference to single embolized rats is evaluated using the nonparametric Mann-Whitney test. **c) Mesenteric blood flow.** The mesenteric blood flow was investigated at the end of experiment. The results are shown in ml/min/100g/kg body weight. The mesenteric blood flow was increased significantly in weekly embolized rats compared to single embolized and control rats. **d) Mesenteric shunt volume.** The mesenteric shunt volume was assessed at the end of experiment. The results of weekly, single embolized and control rats are shown in ml/min/g liver. The mesenteric shunt volume was increased most in weekly embolized rats and less in single embolized rats. In control rats the mesenteric shunt volume was lowest. **e) Splanchnic vascular resistance.** At the end of experiments the splanchnic vascular resistance was assessed using the coloured microsphere technique. The splanchnic vascular resistance was significantly decreased after weekly embolization in rats. The results of are shown in mmHg/min/100g/ml. *p<0.05 / **p<0.005 / ***p<0.0008 vs. Control; #p<0.05 vs. single embolization.

Every week the PP was measured in all rat groups. After the embolization of microspheres, the PP increased continuously every week (weekly embolization group). After a single embolization of microspheres the PP did not change (single embolization group). In control rats, which were not embolized with microspheres, the PP did not change (Fig 3a).

### Portal pressure in NCIPH rats at end of experiments

At the end of experiments, after 3 weeks, the PP was significantly increased in weekly embolized rats compared to single embolized rats (Fig 3b). The mesenteric blood flow was significantly increased after weekly embolization compared to single embolized and control rats (Fig 3c). Also the mesenteric shunt volume was significantly increased after weekly embolization compared to control rats (Fig 3d). Splanchnic vascular resistance was significantly decreased in weekly embolized rats compared to rats with only a single embolization (Fig 3e). This is in line with hemodynamic findings in portal hypertension [16,25]. There were no significant differences in the MAP (weekly embolization: 87.0 ± 13.2; single embolization: 96.2 ± 12.2; control: 91.0 ± 6.9) and in the cardiac output (weekly embolization: 19.0 ± 1.6; single embolization: 29.8 ± 5.3; control: 16.7 ± 4.1), respectively.

### Comparison of human and rat data

In summary the features that we find in humans could be reproduced in our new model of NCIPH (Table 2).

**Table 2:** Characteristics of NCIPH human and weekly embolized rats. Summary of the characteristics found in human NCIPH patients and in the novel rat model mimicking NCIPH. Portal hypertension, Fibrosis, activation of myofibroblasts as well as atrophia and hyperplasia were present in human NCIPH patients and also in the rat model of NCIPH.

Characteristics	Human NCIPH	Weekly embolized rats
Presence of portal hypertension	+	+
Fibrosis	+	+
Activation of myofibroblast	+	+
Atrophia / Hyperplasia	+	+

## Discussion

The present study is the first study to offer an animal model of NCIPH, which compares the findings in rats with the findings in patients with NCIPH.

NCIPH is a rare disorder and causes substantial complications such as gastro-abdominal bleeding [7]. Causes and pathophysiology of NCIPH are still unknown [4]. However, various possible associations, e.g. micro-thrombotic lesions, have been described so far.

A distinctive histopathological feature of NCIPH is obliteration of small portal venules (occlusive venopathy) that may lead to impairment in the intrahepatic portal perfusion resulting parenchymal atrophy, marked by decreased size of cytoplasma and condensed nucleus this was clearly visible in HE stainings. Nodular regenerative hyperplasia was defined by micronodular transformation of the liver parenchyma, with central hyperplasia, an atrophic rim, and no fibrosis. Indeed, in specimens of liver biopsies, a major number of fibrotic processes and abnormal vascularization were shown in our patients, which could be reproduced in our animal models (presence of portal hypertension, fibrosis, activation of myofibroblast, atrophia or hyperplasia).

Repetitive *in vivo* thrombotic events have been proposed as a potential cause for the development of NCIPH. This occlusion by microspheres might serve as a model in animals. In line with this hypothesis, one of our patients had been treated with antiplatelet medication and showed an important improvement in bilirubin, creatinine and platelet count in follow up.

Indeed, using repetitive weekly embolization, PP increased significantly at each time point of embolization in rats. This experiment confirms that our animal model can reproduce the proposed pathogenesis of NCIPH by micro-thrombotic insults. As expected, portal hypertension induced by repetitive embolization in the portal vein was combined with increased mesenteric blood flow and mesenteric shunt volume, suggesting that the full picture of portal hypertension with hyperdynamic circulation was developed. Also, liver histology and intrahepatic fibrosis showed, that our model correctly reflects HSC activation and discrete periportal fibrosis accumulation, which has been observed in patients with NCIPH [3], and were discussed as an important pathogenic stress for the development of NCIPH [10]. Therefore, we offer a model for the development of NCIPH, which may help to test strategies for the improvement of portal hypertension and complications of NCIPH.

Histological changes observed in our animal model nicely correspond to changes found in human livers of patients with NCIPH, as described previously [2,3]. Importantly, in our patients liver biopsy could confirm the presence of fibrosis accumulation and HSC activation, which was induced by repeated micro-thrombotic events in the animal model.

The main difference between this model and other models of portal hypertension is that in the majority of the models liver cirrhosis is induced to elicit portal hypertension, while in this model liver does not exhibit cirrhosis. Besides immunological disorders, chronic infections, exposures to medications or toxins and genetic disorders, prothrombotic conditions also lead to NCIPH. NCIPH patients have a high incidence of portal vein thrombosis. A model of portal vein thrombosis with consecutive portal hypertension is Partial Portal Vein Ligation (PPVL), which is not eligible for our study, since portal vein thrombosis is not the reason but the consequence of NCIPH.

Certainly the rat model has its limitations. First, it is a technical challenge to perform invasive PP measurements and embolization *in vivo*. Another potential limitation of this model is that patients with NCIPH are heterogeneous concerning their underlying etiology, which comprises immunological disorders or HIV. In contrast, NCIPH in the rats was induced by surgery and iatrogenic embolization. Despite such short comings, the good correspondence of histological findings between patients with NCIPH and rats as well as induction of portal hypertension in the absence of cirrhosis suggests that repeated experimental micro-embolization at least reproduce key findings of NCIPH. Of note, our results suggest that a single thrombotic event is not sufficient but repeated portal venous micro-thrombotic events are required to induce NCIPH. Thus, our model may allow to study therapeutic interventions of various stages of the pathogenic process ultimately resulting in NCIPH.

## Abbreviations

AEC: 3-amino-9-ethylcarbazole
αSMA: α-smooth muscle actin
GADPH: Glyceraldehyde 3-phosphate dehydrogenase
HSC: Hepatic stellate cells
IHC: Immunohistochemistry
i.p.: intraperitoneal
MAP: Mean arterial pressure
NCIPH: non-cirrhotic idiopathic portal hypertension
NRH: nodular regenerative hyperplasia
NCPF: non-cirrhotic Portal Fibrosis
PE: Polyethylene
PP: portal pressure
s. c.: subcutaneous
